# Supplementary material for: Quantification of aminobutyric acids and their clinical applications as biomarkers for osteoporosis
Source: Commun Biol. 2020 Jan 22;3:39. doi: 10.1038/s42003-020-0766-y (PMC6976694; doi:10.1038/s42003-020-0766-y)
Supplement: Supplementary file 1 — Supplementary Figures and Tables [file 42003_2020_766_MOESM1_ESM.pdf]

Supplementary Data:

Supplementary Figure 1

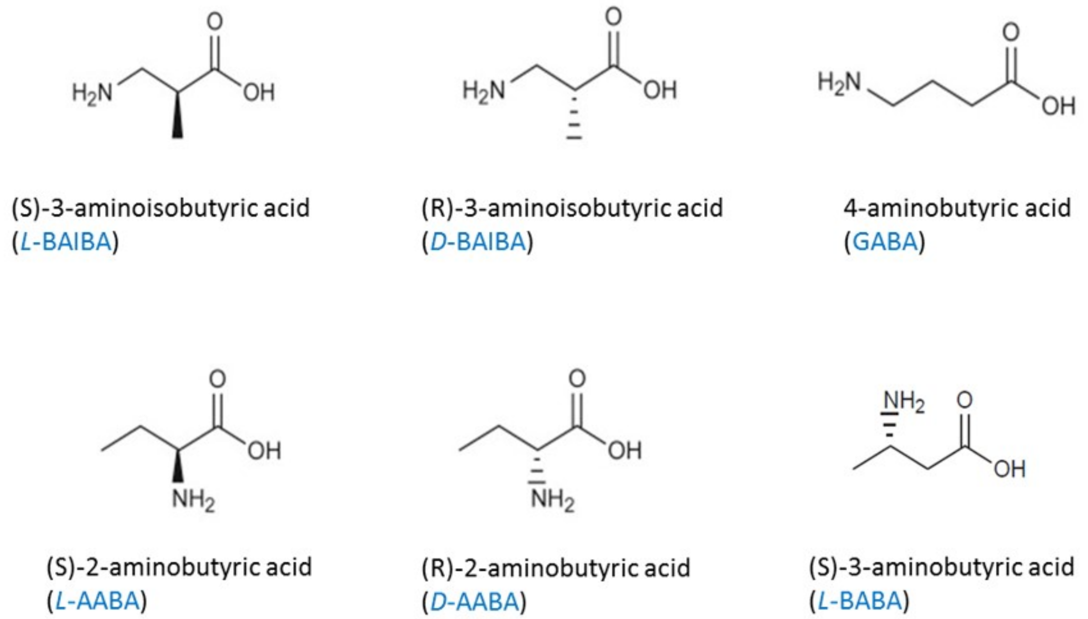

Supplementary Fig. 1. Structures of the six major isomeric aminobutyric acids identified and chromatographically separated using our new LC-MS/MS method.

Supplementary Figure 2

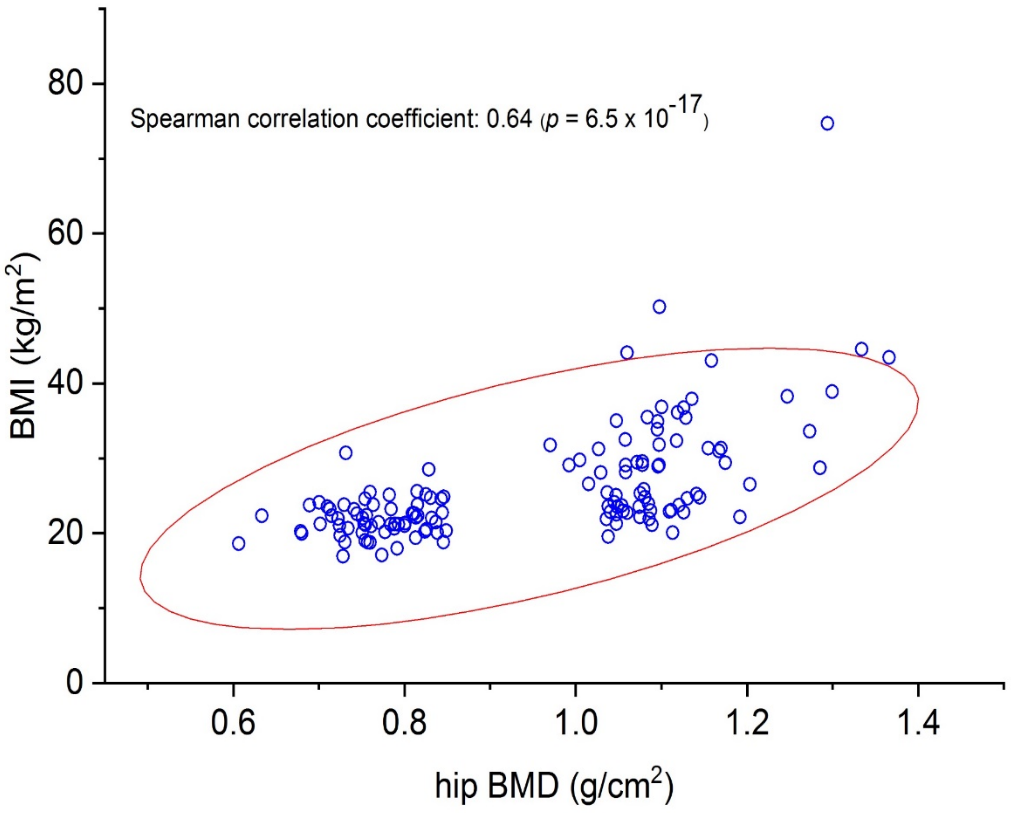

Supplementary Fig. 2. Association analysis between BMD and BMI in young Caucasian women subjects. n=136.

Supplementary Figure 3

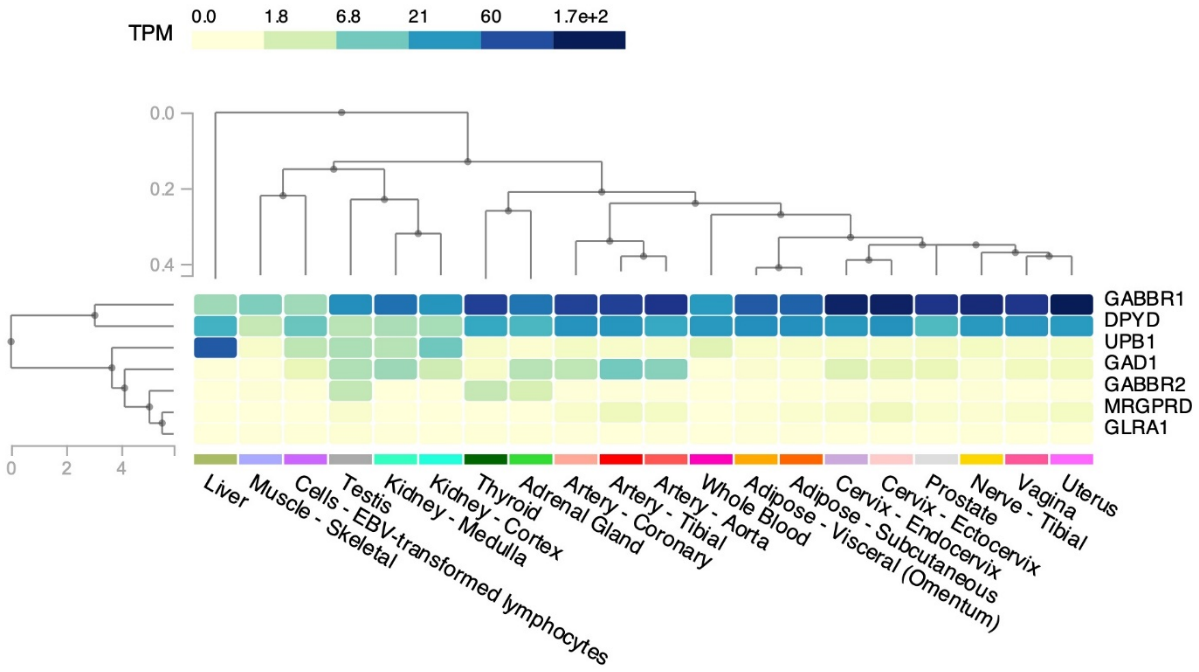

Supplementary Fig. 3. **A heatmap for reference panel of the median TPM gene expression levels for the aminobutyric acids related genes.** The tissues were selected by either potential bone-related, sex-related tissues, or relatively high expressed of the genes. The reference expression profiles for the tissues were obtained from GTXs Portal (https://www.gtexportal.org/home/) on October 30, 2019.

TPM: Transcripts per million.

Supplementary Table 1. Analytes and deuterated standards and their MRM transition conditions

#	Compounds	Retention time (min)	MRM (+/-)	MRM Transition (*m/z*)	MS parameters			Compound type
					Q1(V)	CE(V)	Q3(V)	
1	*L*-AABA	4.8	MRM(+)	104.1>58.0	-31	-19	-26	Analyte
2	*D*-AABA	7.4	MRM(+)	104.1>58.0	-31	-19	-26	Analyte
3	*L*-BABA	8.4	MRM(+)	104.1>86.0	-30	-18	-25	Analyte
4	*D*-BAIBA	10.2	MRM(+)	104.1>86.0	-30	-18	-28	Analyte
5	*L*-BAIBA	11.3	MRM(+)	104.1>86.0	-30	-17	-28	Analyte
6	GABA	15.8	MRM(+)	104.1>69.0	-29	-12	-16	Analyte
7*	*L*-AABA-d_6	4.8	MRM(+)	110.1>64.0	-11	-24	-25	IS
8*	*D*-AABA-d_6	7.4	MRM(+)	110.1>64.0	-11	-24	-25	IS
9*	*D*-BAIBA-d_3	10.2	MRM(+)	107.1>89.0	-30	-15	-18	IS
10*	*L*-BAIBA-d_3	11.3	MRM(+)	107.1>89.0	-30	-15	-18	IS
11*	GABA-d_6	15.8	MRM(+)	110.1>92.0	-28	-12	-19	IS

*deuterated standards. IS, internal standard.

Supplementary Table 2. Slopes and coefficients (R^2) of linear regression analysis for aminobutyric acid isomers spiked in 5% BSA in PBS (w/v) versus in plasma, serum and CSF (0.02 – 163.84 µM) to assess the parallelism of their matrix effects.

	**Human Serum**			**Mouse Serum**			**Mouse Plasma**			**Human CSF**		
	**Linear range (µM)**	**Slope**	**R^2**	**Linear range (µM)**	**Slope**	**R^2**	**Linear range (µM)**	**Slope**	**R^2**	**Linear range (µM)**	**Slope**	**R^2**
***L*-BAIBA**	0.02 - 40.96	0.90	0.9986	0.02 - 40.96	0.88	0.9957	0.02 - 40.96	0.93	0.9995	0.02 - 20.48	0.98	0.9995
***D*-BAIBA**	0.02 - 40.96	0.94	0.9952	0.02 - 40.96	0.92	0.9957	0.02 - 40.96	0.93	0.9996	0.02 - 20.48	0.96	0.9997
**GABA**	0.02 - 40.96	0.91	0.9905	0.02 - 40.96	0.94	0.9976	0.02 - 40.96	0.90	0.9995	0.02 – 20.48	0.96	0.9991
***L*-AABA**	0.16 - 163.84	0.96	0.9977	0.16 - 163.84	0.87	0.9869	0.16 - 163.84	0.99	0.9942	0.16 - 163.84	1.04	0.9914
***D*-AABA**	0.16 - 163.84	1.07	0.9960	0.16 - 163.84	1.12	0.9915	0.16 - 163.84	1.10	0.9963	0.16 - 163.84	1.06	0.9979

CSF, cerebrospinal fluid.

Supplementary Table 3. Background and demographics of the studied women without (Control) or with (CASE) osteoporotic fractures (48-80 years old) and measured serum concentrations of isomeric aminobutyric acids.

Characteristics		All	CASE	Control
**Age** (years), mean ± SD		60.59 ± 6.55	61.00 ± 6.77	60.51 ± 6.46
48-59, n (%)		26 (48.1)	13 (48.1)	13 (48.1)
60-80, n (%)		28 (51.9)	14 (51.9)	14 (51.9)
**Gender**				
Female, n (%)		54 (100)	27 (100)	27 (100)
Male, n (%)		0 (0)	0 (0)	0 (0)
**T-score**				
TSpine, mean ± SD		-1.15 ± 0.83	-1.24 ± 0.86	-1.07 ± 0.80
THIP, mean ± SD		-0.92 ± 0.70	-0.92 ± 0.68	-0.92 ± 0.73
**Location of fractures**				
= 0, n (%)		27 (50)	0 (0)	27 (100)
= 1, n (%)		8 (14.8)	8 (29.6)	0 (0)
= 2, n (%)		10 (18.5)	10 (37.0)	0 (0)
≥ 3, n (%)		9 (16.7)	9 (33.3)	0 (0)
Axial fractures		9 (16.7)	9 (33.3)	0 (0)
Appendicular fractures		18 (33.3)	18 (66.7)	0 (0)
**Serum concentrations** (µM)				
L-BAIBA	mean ± SD	N.D.	N.D.	N.D.
	Range	-	-	-
D-BAIBA	mean ± SD	1.13 ± 0.46	1.07 ± 0.45	1.20 ± 0.46
	Range	0.38–2.24	0.38–1.97	0.71–2.24
GABA	mean ± SD	0.13 ± 0.03	0.12 ± 0.02	0.13 ± 0.03
	Range	0.08–0.20	0.08–0.16	0.09–0.20
L-AABA	mean ± SD	4.78 ± 1.44	4.82 ± 1.43	4.75 ± 1.46
	Range	2.31–7.86	2.31–7.78	2.36–7.86
D-AABA	mean ± SD	N.D.	N.D.	N.D.
	Range	-	-	-

N.D., not determined.

Supplementary Table 4. Background and demographics of the younger women group (21 - 41 years old) with various bone mineral density (BMD) and measured serum concentrations of isomeric aminobutyric acids.

**Characteristics**		**Mean ± SD**	**Minimum**	**Maximum**	**Sample size**
**Age** (years)		31.5 ± 5.1	21	40.6	136
**Gender**					
Female, n (%)	136 (100)				
Male, n (%)	0 (0)				
**Weight** (kg)		70.3 ± 21.4	44.5	220	136
**Height** (cm)		164.6 ± 6.4	150	182.6	136
**BMI** (kg m^{-2})		26.0 ± 7.5	17	74.7	136
**Waist Circumference** (cm)		78.1 ± 16.1	41.5	132	136
**T-score** (hip)		0.03 ± 1.49	-2.75	3.48	136
**HTOT_BMD** (cm^{-2})		0.95 ± 0.18	0.606	1.366	136
**Smoke**, n (%)	48 (35)				
**Drinking**, n (%)	104 (76)				
**Pure Alcohol Intake** (ml)		36.5 ± 52.4	0	336	136
**Physical Activity** (times/week)		3.1 ± 2.2	0	7	136
**Dairy Intake** (cups per day)*		1.6 ± 1.3	0	8	136
**Serum concentrations** (µM)					
L-BAIBA		0.115 ± 0.030	0.076	0.157	6
D-BAIBA		1.98 ± 0.93	0.58	6.20	135
GABA		0.42 ± 0.12	0.21	0.74	135
L-AABA		31.82 ± 13.53	4.48	79.72	135
D-AABA		N.D.	N.D.	N.D.	136

*Diary Intake (cups per day): three different types of the dairy intake have been converted to the same unit. 1) The milk was measured by cup unit; 2) Yogurt: 8 ounces = 1 cup; 3) Cheese: 1.5 ounces = 1 cup. Therefore, Total Dairy Intake = cheese cups per day + yogurt cups per day + milk cups per day.

N.D., not determined.

Supplementary Table 5. The list of GWAS summary statistics for VEGAS gene-based analysis

Short Study	Traits	Data Usage	Ref
GEFOS2	FN-BMD<br>LS-BMD	Provided both pooled and gender-stratified summary statistics.	[1]
GEFOS ALLFX	Fracture		[2]
GEFOS LIFE COURSE	Total body BMD	Provided both pooled and age-stratified summary statistics. Five age strata, each spanning 15 years, including “less 15”, “15 to 30”, “30 to 45”, “45 to 60”, and “60 more”.	[3]
UKBB 2017	eBMD		[4]
UKBB 2018	eBMD<br>Fracture	The p-values from infinitesimal model were used for fracture data, and the p-values from non-infinitesimal model were used for eBMD data.	[5]

BMD: bone mineral density; eBMD: Bone mineral density estimated from quantitative heel ultrasounds,

FB BMD: Femoral Neck BMD, LS-BMD: Lumbar Spine BMD.

Supplementary Table 6. Genes coding for enzymes and receptors associated with BAIBA and GABA metabolism.

**Aminobutyric Acids**	**Symbol**	**Description**	**Ref.**
BAIBA	*AGXT2*	Alanine--Glyoxylate Aminotransferase 2	6
	FFAR3[*]	G protein-coupled receptor 41	7,8
	MRGPRD[%]	MAS Related GPR Family Member D	9
	DPYD[#]	Dihydropyrimidine Dehydrogenase	10,11
	DPYS	Dihydropyrimidinase	12
	UPB1	Beta-Ureidopropionase 1	13
	ALDH6A1	Methylmalonate-Semialdehyde Dehydrogenase	14
L-BAIBA	*ABAT*	4-Aminobutyrate Aminotransferase	15
D-BAIBA	*AGXT2*	Alanine--Glyoxylate Aminotransferase 2	16
GABA	*GABBR1*	Gamma-Aminobutyric Acid Type B Receptor Subunit 1	17
	GABBR2	Gamma-Aminobutyric Acid Type B Receptor Subunit 2	17
	GLRA1	Glycine receptor subunit alpha-1	18
	GLRA2[*]	Glycine receptor subunit alpha-2	19
	GLRA3	Glycine receptor subunit alpha-3	20
	GLRA4[*]	Glycine receptor subunit alpha-4	21
	GAD1	Glutamate Decarboxylase 1	22
	ABAT	4-Aminobutyrate Aminotransferase	23

[*] No summary statistics from all GWAS studies, [%] No summary statistics in GEFOS ALLFX and GEFOS2 studies, [#] No summary statistics in UKBB2017 and UKBB2018 studies.

Supplementary Table 7. Background and demographics of the 122 younger women group (21 - 41 years old) for which bllod was used for transcriptomics analysis.

Characteristics		Mean ± SD	Minimum	Maximum	Sample size
**Age** (years)		31.5 ± 5.1	21	40.6	122
**Gender**					
Female, n (%)	122 (100)				
Male, n (%)	0 (0)				
**Weight** (kg)		70.8 ± 22.0	44.5	220	122
**Height** (cm)		164.6 ± 6.5	150	182.6	122
**BMI** (kg m^{-2})		25.8 ± 6.4	17	50.2	122
**Waist Circumference** (cm)		78.8 ± 16.2	48.5	132	122
**T-score** (hip)		0.01 ± 1.5	-2.75	3.48	122
**HTOT_BMD** (cm^{-2})		0.9 ± 0.20	0.6065	1.366	122
**Smoke**, n (%)	46 (38)				
**Drinking**, n (%)	93 (76)				
**Pure Alcohol Intake** (ml)		37.0 ± 54.7	0	336	122
**Physical Activity** (times/week)		3.1 ± 2.2	0	7	122
**Dairy Intake** (cups per day)*		1.55 ± 1.3	0	8	122
**Serum concentrations** (µM)					
L-BAIBA		0.115 ± 0.030	0.076	0.157	6
D-BAIBA		1.98 ± 0.9	0.64	6.20	122
GABA		0.42 ± 0.12	0.21	0.74	122
L-AABA		32.33 ± 13.92	4.48	79.72	122
D-AABA		N.D.	N.D.	N.D.	122

*Diary Intake (cups per day): three different types of the dairy intake have been converted to the same unit. 1) The milk was measured by cup unit; 2) Yogurt: 8 ounces = 1 cup; 3) Cheese: 1.5 ounces = 1 cup. Therefore, Total Dairy Intake = cheese cups per day + yogurt cups per day + milk cups per day.

N.D., not determined.

Supplementary Table 8. Measured expression levels of aminobutyric acids-related genes in human peripheral blood monocytes.

Subject ID	Expression levels (TPM)						
	DPYD	***GABBR1***	***GABBR2***	***GAD1***	***GLRA1***	***MRGPRD***	***UPB1***
DNAM01700	98.18	83.17	0	0	0.44	0	0.35
DNAM01701	173.85	67.85	0	0	0	0	0
DNAM01702	169.45	69.26	0	0	0	0	0
DNAM01703	165.22	42.33	0	0.05	0	0	0.28
DNAM01704	283.61	101.49	0	0	0.34	0	0.27
DNAM01705	149.44	38.44	0	0.07	0	0	0.38
DNAM01706	280.39	29.74	0.02	0	0.08	0	0.33
DNAM01707	244.34	34.6	0	0.75	0.28	0	0.33
DNAM01708	114.03	132.82	0	0	0	0.28	0.12
DNAM01709	48.6	56.85	0	0	0	0	0
DNAM01710	285.6	36.87	0	0	0	0.31	0
DNAM01711	21.94	55.87	0	0	0	0	0
DNAM01712	215.7	53.14	0	0	0	0	0.25
DNAM01714	134.12	73.62	0	0	0.13	0.27	0.69
DNAM01715	93.19	41.21	0	0	0	0	0.41
DNAM01716	61.72	55.07	0	0	0	0	0
DNAM01719	90.95	64.24	0	0	0	0	1.04
DNAM01720	110.44	67.4	0	0	0	0	1.44
DNAM01722	302.85	43.39	0	0	0.23	0	0.61
DNAM01723	190.08	87.22	0	0.08	0.17	0.31	0.14
DNAM11001	29.55	5.04	0	0	0	0	0
DNAM11002	24.07	6.63	0	0	0	0	0
DNAM11005	48.91	9.94	0	0	0	0	0.24
DNAM11006	98.34	28.53	0	0	0	0	0.28
DNAM11007	202.35	31.04	0	0	0	0	0.19
DNAM11008	55.37	19.86	0	0	0.19	0	0.31
DNAM11009	94.47	41.4	0	0	0.22	0	0.18
DNAM11010	98.01	31.44	0	0	0	0	0
DNAM11012	127.74	18.44	0	0	0	0	0.37
DNAM11013	5.74	1.69	0	0	0	0	0
DNAM11014	97.44	30.69	0	0	0	0	0.16
DNAM11015	85.56	28	0	0	0	0	0.86
DNAM11016	124.06	39.83	0	0	0	0	0.29
DNAM11017	100.2	68.2	0	0	0	0	0

DNAM11018	28.84	10.79	0	0.3	0	0	0
DNAM11019	23.72	9.78	0	0.24	0	0	0.56
DNAM11020	188.3	13.31	0	0	0.39	0	0.21
DNAM11021	140.48	38.74	0	0	0.44	0	0.57
DNAM11027	5.16	3.62	0	0	0	0	0.13
DNAM11030	70.56	27.27	0	0	0.32	0.23	0.3
DNAM11031	230.42	34.34	0	0	0.17	0	0.42
DNAM11032	242.07	15.19	0	0	0	0	0.5
DNAM11033	178.49	44.89	0	0	0	0	0.08
DNAM11037	231.04	14.95	0	0	0.12	0	0.86
DNAM11038	203.74	2.94	0	0	0.12	0	0.49
DNAM11039	223.48	10.34	0	0	0	0	0.08
DNAM11040	187.47	13.75	0	0.04	0	0	0.08
DNAM11041	151.03	63.1	0	0	0.18	0	0.14
DNAM11042	135.09	20.78	0	0.07	0.16	0.29	0.75
DNAM11045	233.18	41.52	0	0	0	0.14	0.06
DNAM11046	332.31	79.02	0	0	0	0	0.76
DNAM11047	203.91	58.95	0	0	0	0	0.35
DNAM11048	269.18	56.96	0	0	0	0	0.15
DNAM11049	183.39	10.55	0	0	0	0	0.46
DNAM11050	176.87	10.16	0.11	0	0	0	0
DNAM11051	241.72	24.19	0	0	0	0	0.59
DNAM11054	337.63	135.96	0	0.12	0	0	0.61
DNAM11055	104.26	43.92	0	0	0	0	0.53
DNAM11056	245.97	75.44	0	0.17	0.23	0	0.2
DNAM11057	234.58	31.31	0	0.09	0	0	0.31
DNAM11058	39.02	18.3	0	0	0	0	0
DNAM11060	151.99	16.51	0	0	0.11	0	0
DNAM11061	176.96	26.61	0	0	0.16	0	0.32
DNAM11064	1.7	12.83	0	0	0	0	0.3
DNAM11065	234.57	23.85	0	0	0	0	0
DNAM11066	163.18	26	0	0	0.11	0	0.09
DNAM11068	195.49	32.4	0.03	0	0	0.18	0.85
DNAM11069	22	145.85	0.17	0	0	0	0
DNAM11071	96.53	41.18	0	0	0	0	0.13
DNAM11072	102.17	13.73	0	0	0.09	0	0.15
DNAM11074	229.48	54.19	0	0	0	1.02	1.03
DNAM11075	0.12	19.97	0	0	0	0	0
DNAM11076	205.28	56.64	0	0.49	0.12	0	0.38
DNAM11077	113.48	22.22	0	0	0	0	0

DNAM11078	235.58	29.7	0	0.05	0	0	0.42
DNAM11079	199.61	26.93	0	0.15	0.3	0	0.8
DNAM11080	208.94	34.3	0	0	0	0	0.38
DNAM11081	214.22	75	0	0	0.07	0	0.58
DNAM11083	2.39	17.27	0	0	0	0	0
DNAM11084	224.84	25.03	0	0	0.1	0	0.48
DNAM11085	249.47	22.68	0	0	0	0	0.09
DNAM11086	0.52	59.51	0	0	0	0	0
DNAM11087	96.49	100.09	0	0	0	0	0
DNAM11088	210.65	37.66	0	0	0.11	0	0.5
DNAM11089	195.58	11.45	0	0	0.11	0	0.39
DNAM11090	9.5	101.17	0	0	0	0	0
DNAM11091	157.71	10.23	0	0	0	0	0.48
DNAM11092	2.16	48.17	0	0	0	0	0
DNAM11093	171.07	41.8	0	0	0	0	0.61
DNAM11094	268.26	24.28	0	0	0	0	0.36
DNAM11095	53.46	19.56	0	0	0	0	0
DNAM11096	257.85	19.8	0	0	0	0	0.59
DNAM11097	238.38	30.99	0.04	0	0	0.29	0.5
DNAM11098	174.51	21.76	0	0	0	0	0.09
DNAM11099	149.25	76.4	0	0.96	0	0	0
DNAM11100	52.09	171.45	0	0	0	0	0
DNAM11101	207.84	46.49	0	0	0	0	0.1
DNAM11102	264.03	13.66	0	0	0	0	0.55
DNAM11103	236.47	8.01	0	0	0	0	1.11
DNAM11104	152.56	32.69	0	0	0	0	0.34
DNAM11105	13.61	138.89	0	0	0	0	0
DNAM11108	150.19	8.64	0	0.16	0	0	0.21
DNAM11110	163.63	10.51	0	0	0	0	0
DNAM11111	146.24	22.72	0	0	0	0	0.1
DNAM11114	174.44	18.31	0	0.18	0	0	0.43
DNAM11116	247.33	7.87	0.02	0	0.08	0	0
DNAM11117	70.56	1.12	0	0	0	0	0
DNAM11118	91.4	9.79	0	0	0	0	0.99
DNAM11119	101.2	16.35	0	0.04	0	0	0.3
DNAM11120	226.46	12.67	0.07	0	0	0	0.32
DNAM11121	257.56	9.72	0	0	0	0	0.21
DNAM11122	229.49	16.98	0	0.07	0	0	0.51
DNAM11123	222.26	99.6	0	0	0.12	0	0.19
DNAM11124	231.74	32.53	0	0	0.16	0	0.13

DNAM11125	212.17	30.95	0	0	0	0	0.13
DNAM11126	171.08	36.09	0	0.42	0	0	0.85
DNAM11127	257.78	28.28	0	0	0	0	0.52
DNAM11128	251.12	31.57	0	0.04	0	0	0.59
DNAM11129	252.02	56.6	0	0	0	0	1.08
DNAM11130	170.66	30.16	0	0.06	0	0	0.11
DNAM11131	241.73	49.08	0	0	0	0	0.34
DNAM11132	46.2	50.16	0.04	0	0	0.76	1.33

TPM, Transcripts per million.

References

1. Estrada, K., *et al.* Genome-wide meta-analysis identifies 56 bone mineral density loci and reveals 14 loci associated with risk of fracture. *Nat Genet* **44**, 491-501 (2012).
2. Trajanoska, K., *et al.* Assessment of the genetic and clinical determinants of fracture risk: genome wide association and mendelian randomisation study. *BMJ* **362**, k3225 (2018).
3. Medina-Gomez, C., *et al.* Life-Course Genome-wide Association Study Meta-analysis of Total Body BMD and Assessment of Age-Specific Effects. *Am J Hum Genet* **102**, 88-102 (2018).
4. Kemp, J.P., *et al.* Identification of 153 new loci associated with heel bone mineral density and functional involvement of GPC6 in osteoporosis. *Nat Genet* **49**, 1468-1475 (2017).
5. Morris, J.A., *et al.* An atlas of genetic influences on osteoporosis in humans and mice. *Nat Genet* **51**, 258-266 (2019).
6. Kittel, A., *et al.* Alanine-glyoxylate aminotransferase 2 (AGXT2) polymorphisms have considerable impact on methylarginine and beta-aminoisobutyrate metabolism in healthy volunteers. *PLoS One* **9**, e88544 (2014).
7. Roberts, L.D., *et al.* beta-Aminoisobutyric acid induces browning of white fat and hepatic beta-oxidation and is inversely correlated with cardiometabolic risk factors. *Cell Metab* **19**, 96-108 (2014).
8. Brown, A.J., *et al.* The Orphan G protein-coupled receptors GPR41 and GPR43 are activated by propionate and other short chain carboxylic acids. *J Biol Chem* **278**, 11312-11319 (2003).
9. Kitase, Y., *et al.* beta-aminoisobutyric Acid, l-BAIBA, Is a Muscle-Derived Osteocyte Survival Factor. *Cell Rep* **22**, 1531-1544 (2018).
10. van Kuilenburg, A.B., Stroomer, A.E., Abeling, N.G. & van Gennip, A.H. A pivotal role for beta-aminoisobutyric acid and oxidative stress in dihydropyrimidine dehydrogenase deficiency? *Nucleosides Nucleotides Nucleic Acids* **25**, 1103-1106 (2006).
11. Cretton, E.M., *et al.* In vitro and in vivo disposition and metabolism of 3'-deoxy-2',3'-didehydrothymidine. *Antimicrob Agents Chemother* **37**, 1816-1825 (1993).
12. van Kuilenburg, A.B., Stroomer, A.E., Bosch, A.M. & Duran, M. Beta-alanine and beta-aminoisobutyric acid levels in two siblings with dihydropyrimidinase deficiency. *Nucleosides Nucleotides Nucleic Acids* **27**, 825-829 (2008).
13. van Kuilenburg, A.B., *et al.* beta-Ureidopropionase deficiency: an inborn error of pyrimidine degradation associated with neurological abnormalities. *Hum Mol Genet* **13**, 2793-2801 (2004).

14. Marcadier, J.L., *et al.* Mutations in ALDH6A1 encoding methylmalonate semialdehyde dehydrogenase are associated with dysmyelination and transient methylmalonic aciduria. *Orphanet J Rare Dis* **8**, 98 (2013).
15. Kupiecki, F.P. & Coon, M.J. The enzymatic synthesis of beta-aminoisobutyrate, a product of valine metabolism, and of beta-alanine, a product of beta-hydroxypropionate metabolism. *J Biol Chem* **229**, 743-754 (1957).
16. Kontani, Y., Kaneko, M., Kikugawa, M., Fujimoto, S. & Tamaki, N. Identity of D-3-aminoisobutyrate-pyruvate aminotransferase with alanine-glyoxylate aminotransferase 2. *Biochim Biophys Acta* **1156**, 161-166 (1993).
17. Kaupmann, K., *et al.* GABA(B)-receptor subtypes assemble into functional heteromeric complexes. *Nature* **396**, 683-687 (1998).
18. Yang, Z., Sun, G., Yao, F., Tao, D. & Zhu, B. A novel compound mutation in GLRA1 cause hyperekplexia in a Chinese boy- a case report and review of the literature. *BMC Med Genet* **18**, 110 (2017).
19. McCracken, L.M., *et al.* Glycine receptor alpha3 and alpha2 subunits mediate tonic and exogenous agonist-induced currents in forebrain. *Proc Natl Acad Sci U S A* **114**, E7179-E7186 (2017).
20. Nikolic, Z., *et al.* The human glycine receptor subunit alpha3. Glra3 gene structure, chromosomal localization, and functional characterization of alternative transcripts. *J Biol Chem* **273**, 19708-19714 (1998).
21. Simon, J., Wakimoto, H., Fujita, N., Lalande, M. & Barnard, E.A. Analysis of the set of GABA(A) receptor genes in the human genome. *J Biol Chem* **279**, 41422-41435 (2004).
22. Magri, C., *et al.* A novel homozygous mutation in GAD1 gene described in a schizophrenic patient impairs activity and dimerization of GAD67 enzyme. *Sci Rep* **8**, 15470 (2018).
23. Medina-Kauwe, L.K., *et al.* 4-Aminobutyrate aminotransferase (GABA-transaminase) deficiency. *J Inherit Metab Dis* **22**, 414-427 (1999).
