# Supplementary material for: Quantification of aminobutyric acids and their clinical applications as biomarkers for osteoporosis
Source: Commun Biol. 2020 Jan 22;3:39. doi: 10.1038/s42003-020-0766-y (PMC6976694; doi:10.1038/s42003-020-0766-y)
Supplement: Supplementary file 3 — Reporting summary (New) [file 42003_2020_766_MOESM3_ESM.pdf]

nature research

Corresponding author(s): Marco Brotto

Last updated by author(s): Jan 8, 2020

Reporting Summary

Nature Research wishes to improve the reproducibility of the work that we publish. This form provides structure for consistency and transparency in reporting. For further information on Nature Research policies, see Authors & Referees and the Editorial Policy Checklist.

Statistics

For all statistical analyses, confirm that the following items are present in the figure legend, table legend, main text, or Methods section.

n/a	Confirmed	
☐	☒	The exact sample size (*n*) for each experimental group/condition, given as a discrete number and unit of measurement
☐	☒	A statement on whether measurements were taken from distinct samples or whether the same sample was measured repeatedly
☐	☒	The statistical test(s) used AND whether they are one- or two-sided<br>*Only common tests should be described solely by name; describe more complex techniques in the Methods section.*
☐	☒	A description of all covariates tested
☐	☒	A description of any assumptions or corrections, such as tests of normality and adjustment for multiple comparisons
☐	☒	A full description of the statistical parameters including central tendency (e.g. means) or other basic estimates (e.g. regression coefficient) AND variation (e.g. standard deviation) or associated estimates of uncertainty (e.g. confidence intervals)
☐	☒	For null hypothesis testing, the test statistic (e.g. *F*, *t*, *r*) with confidence intervals, effect sizes, degrees of freedom and *P* value noted<br>*Give P values as exact values whenever suitable.*
☒	☐	For Bayesian analysis, information on the choice of priors and Markov chain Monte Carlo settings
☒	☐	For hierarchical and complex designs, identification of the appropriate level for tests and full reporting of outcomes
☐	☒	Estimates of effect sizes (e.g. Cohen's *d*, Pearson's *r*), indicating how they were calculated

Our web collection on statistics for biologists contains articles on many of the points above.

Software and code

Policy information about availability of computer code

Data collection	n/a
Data analysis	The R codes for our analyses are available from the authors on reasonable request, see author contributions for specific data sets.

For manuscripts utilizing custom algorithms or software that are central to the research but not yet described in published literature, software must be made available to editors/reviewers. We strongly encourage code deposition in a community repository (e.g. GitHub). See the Nature Research guidelines for submitting code & software for further information.

Data

Policy information about availability of data

All manuscripts must include a data availability statement. This statement should provide the following information, where applicable:

- Accession codes, unique identifiers, or web links for publicly available datasets
- A list of figures that have associated raw data
- A description of any restrictions on data availability

The data that support the findings of this study are available from the authors on reasonable request due to the nature of these data (human subjects); and its associated privacy and HIPPA regulations. See author contributions for specific data sets. All GWAS summary data for the gene-based association tests have been deposited in the GEFOS website (http://www.gefos.org/?q=documents). The gene expression quantification by RNA-Seq for 122 samples will be made available in dbGaP under accession code phs001960.v1.p1; in the site https://www.ncbi.nlm.nih.gov/projects/gapprev/gap/cgi-bin/study.cgi?study_id=phs001960.v1.p1

Field-specific reporting

Please select the one below that is the best fit for your research. If you are not sure, read the appropriate sections before making your selection.

☒ Life sciences ☐ Behavioural & social sciences ☐ Ecological, evolutionary & environmental sciences

For a reference copy of the document with all sections, see nature.com/documents/nr-reporting-summary-flat.pdf

Life sciences study design

All studies must disclose on these points even when the disclosure is negative.

Sample size	For quantification of serum isomeric aminobutyric acids in human subjects in clinical application, total 136 young women subjects and 54 old women subjects were involved in this study. Detailed information please see the section "Recruitment of human subjects" in manuscript page 23-24.<br>For transcriptomics analysis (RNA-seq) for human peripheral blood monocytes, total 122 subjects were involved in this study. Please see manuscript page 24.<br>For validation of the developed quantification method in this manuscript, we followed guideline from FDA's Bioanalytical Method Development Guidance for Industry. Please see the section "Preparation of standard calibration curves and QC samples" in manuscript page 21-22.
Data exclusions	For method development and validation, no data were excluded from analyses.<br>For comparison analyses of aminobutyric acids in serum samples from human subjects, outliers detected using Grubbs' test (at 0.05 significance level) were excluded.
Replication	For validation of the developed analytical method and its application in different biological fluids, all measures have been taken 5 times to confirm the results.
Randomization	Human participants in this study were recruited by Creighton University Osteoporosis Research Center (ORC), and Louisiana Osteoporosis Study (LOS), respectively. Some factors including lifestyle, medication, etc. were taken into consideration during the recruitment. Please see detailed description in manuscript page 23-24.
Blinding	Yes. Human serum samples from both Tulane and Creighton were shipped to our lab with only basic information i.e. Sample ID, therefore we were blinded to any background and demographics information about these samples we were analyzing. After we sent back the results of serum aminobutyric acids quantification to Tulane and Creighton, detailed demographic information of participants were shared between teams to perform statistical analysis.

Reporting for specific materials, systems and methods

We require information from authors about some types of materials, experimental systems and methods used in many studies. Here, indicate whether each material, system or method listed is relevant to your study. If you are not sure if a list item applies to your research, read the appropriate section before selecting a response.

Materials & experimental systems

n/a	Involved in the study	
☒	☐	Antibodies
☒	☐	Eukaryotic cell lines
☒	☐	Palaeontology
☒	☐	Animals and other organisms
☐	☒	Human research participants
☒	☐	Clinical data

Methods

n/a	Involved in the study	
☒	☐	ChIP-seq
☒	☐	Flow cytometry
☒	☐	MRI-based neuroimaging

Human research participants

Policy information about studies involving human research participants

Population characteristics	Two groups of human subjects were recruited in this study: 1) 54 women (age 48-80 years) with or without single or multiple osteoporotic fractures, and 2) total 136 younger women (age 21 - 41 years) with low or high bone mineral density (BMD) values (hip). Detailed information can be found from the section of "Recruitment of human subjects" in manuscript page 23-24.
Recruitment	1) For the older women group (age 48-80 years) containing both fracturing women and controls, the 54 subjects were recruited by Creighton University Osteoporosis Research Center (ORC) from several approaches. 2) 136 Caucasian females with age between 21-41 years old were recruited from participants in Louisiana Osteoporosis Study (LOS), a cross-sectional study with ongoing recruitment to build a large sample pool (~20,000 subjects) and database for research studies of osteoporosis and other musculoskeletal diseases/traits. Detailed information can be found from section of "Recruitment of human subjects" in

manuscript page 23-24.

Ethics oversight

1) Creighton University Osteoporosis Research Center (ORC) has IRB Approval (Creighton University IRB Committee IRB# 07-14738) for their clinical studies.
2) Louisiana Osteoporosis Study (LOS) has IRB Approval (Biomedical IRB Committee IRB#318016) for their clinical studies.

Note that full information on the approval of the study protocol must also be provided in the manuscript.
